# Bayesian molecular design with a chemical language model

**DOI:** 10.1007/s10822-016-0008-z

**Published:** 2017-03-09

**Authors:** Hisaki Ikebata, Kenta Hongo, Tetsu Isomura, Ryo Maezono, Ryo Yoshida

**Affiliations:** 1 The Graduate University for Advanced Studies (SOKENDAI), Tachikawa, Japan; 2 Japan Advanced Institute of Science and Technology (JAIST), Nomi, Japan; 3 National Institute for Materials Science (NIMS), Tsukuba, Japan; 4 PRESTO, Japan Science and Technology Agency (JST), Kawaguchi, Japan; 5 The KAITEKI Institute, Inc., Tokyo, Japan; 6 The Institute of Statistical Mathematics (ISM), Research Organization of Information and Systems, Tachikawa, Japan

**Keywords:** Inverse-QSPR, Molecular design, Bayesian analysis, Small organic molecules, Natural language processing, SMILES

## Abstract

The aim of computational molecular design is the identification of promising hypothetical molecules with a predefined set of desired properties. We address the issue of accelerating the material discovery with state-of-the-art machine learning techniques. The method involves two different types of prediction; the forward and backward predictions. The objective of the forward prediction is to create a set of machine learning models on various properties of a given molecule. Inverting the trained forward models through Bayes’ law, we derive a posterior distribution for the backward prediction, which is conditioned by a desired property requirement. Exploring high-probability regions of the posterior with a sequential Monte Carlo technique, molecules that exhibit the desired properties can computationally be created. One major difficulty in the computational creation of molecules is the exclusion of the occurrence of chemically unfavorable structures. To circumvent this issue, we derive a chemical language model that acquires commonly occurring patterns of chemical fragments through natural language processing of ASCII strings of existing compounds, which follow the SMILES chemical language notation. In the backward prediction, the trained language model is used to refine chemical strings such that the properties of the resulting structures fall within the desired property region while chemically unfavorable structures are successfully removed. The present method is demonstrated through the design of small organic molecules with the property requirements on HOMO-LUMO gap and internal energy. The R package *iqspr* is available at the CRAN repository.

**Electronic supplementary material:**

The online version of this article (doi:10.1007/s10822-016-0008-z) contains supplementary material, which is available to authorized users.

## Introduction

Computational molecular design has a great potential to promote enormous savings in time and cost in the discovery and development of functional molecules and assembles including drugs, dyes, solvents, polymers, and catalysis. The objective is to computationally create promising molecules that exhibit desired properties of various kinds, simultaneously. For instance, the chemical space of small organic molecules is known to consist of more than $10^{60}$ candidates. The problem entails a considerably complicated multi-objective optimization where it is impractical to fully explore the vast landscape of structure-property relationships. In general, the molecular design process involves two different types of prediction; the forward prediction is aimed at predicting physical, chemical and electric properties of a given molecular structure, and the backward prediction is to inversely identify appropriate molecular structures with the given desired properties. While the former design process is referred to as the quantitative structure-property relationship (QSPR) analysis, the latter is known as the inverse-QSPR analysis [1–9]. In this study, a Bayesian perspective is employed to unify the forward and backward prediction processes. Therefore, the present method is called the Bayesian molecular design.

In cheminformatics or an emerging new research field called materials informatics, there have been extensive studies on the forward prediction; however, there has been considerably less progress made in the backward prediction. An obvious approach to the inverse problem is the use of combinatorial optimization techniques. The objective is to minimize the difference between given desired properties and those attained by the designed molecules. Some previous studies tackled this issue with genetic algorithms (GAs) [2, 4–7, 10–13] and molecular graph enumeration [8, 9, 14]. Graph enumeration is generally less effective due to the combinatorial complexity of the design space. To narrow down the candidates, several ways to use a restricted class of molecular graphs have been investigated [9, 14]. Using GAs [15], which have been more intensively studied, searches for optimal or suboptimal designs by successively modifying chemical structures with genetic operators consisting of mutation, crossover, and selection.

The major difficulty of using a GA lies in the procedure of mutating molecules such that unfavorable structures are successfully excluded, for instance, unfavorable and/or unrealistic chemical bonds such as F–N and C=O=C. This issue is common to the graph enumeration. To avoid the emergence of unfavorable structures, exclusion rules were employed in some studies, particularly those aimed at the design of drug-like molecules [16, 17]. However, such rules might be incomprehensive, and it is impractical to establish a general rule of chemically favorable structures. A promising alternative is fragment assembly methods [4–7, 13, 18–20]. In a structure manipulation step of these methods, randomly chosen substructures are replaced by fragments of existing compounds. While the fragment assembly methods have a certain appeal, as is evident from their widespread use, they suffer from critical disadvantages: (i) the design space is restricted to possible combinations of collected fragments, (ii) the use of a vast amount of fragments entails unacceptably large computational loads to homology search in the fragment exchange operation, and (iii) mutation and crossover operations require computationally intractable graph manipulations. The proposed method circumvents all these issues.

The Bayesian molecular design begins by obtaining a set of machine learning models that forwardly predict properties of a given molecule for multiple design objectives. These forward models are inverted to the backward model through Bayes’ law, combined with a prior distribution. This gives a posterior probability distribution for the backward prediction, which is conditioned by a desired property region. Exploring high-probability regions of the posterior with the Sequential Monte Carlo (SMC) method [21], molecules that exhibit the desired properties are computationally created. The most distinguished feature of this workflow lies in the backward prediction algorithm. In this study, a molecule is described by an ASCII string according to the SMILES chemical language notation. To reduce the emergence of chemically unfavorable structures, a chemical language model is trained, which acquires commonly occurring patterns of chemical substructures by the natural language processing of the SMILES language of existing compounds. The trained model is used to recursively refine SMILES strings of seed molecules such that the properties of the resulting molecules fall in the desired property region while eliminating the creation of unfavorable chemical structures.

The key contributions of the newly proposed method are summarized below.

- *String-based structure refinement* The string representation of molecules enables much faster structure refinements in the backward prediction than those based on graph representation.
- *Generator for chemically favorable structures* The method is designed according to a fragment-free strategy. Structural patterns of known compounds or implied contexts of ‘chemically favorable structures’ are captured by the probabilistic model. Afterward, the resulting SMILES generator will be shown to be very effective in creating chemically plausible hypothetical molecules. The trained model serves as a substitute for a fragment library. This model also forms the prior distribution in the Bayesian analysis.

The forward and backward predictions are pipelined with the R package *iqspr* which is provided through the CRAN repository [22]. The present method is illustrated through the design of small organic molecules exhibiting properties within prescribed ranges of HOMO-LUMO gap and internal energy.

**Fig. 1:**
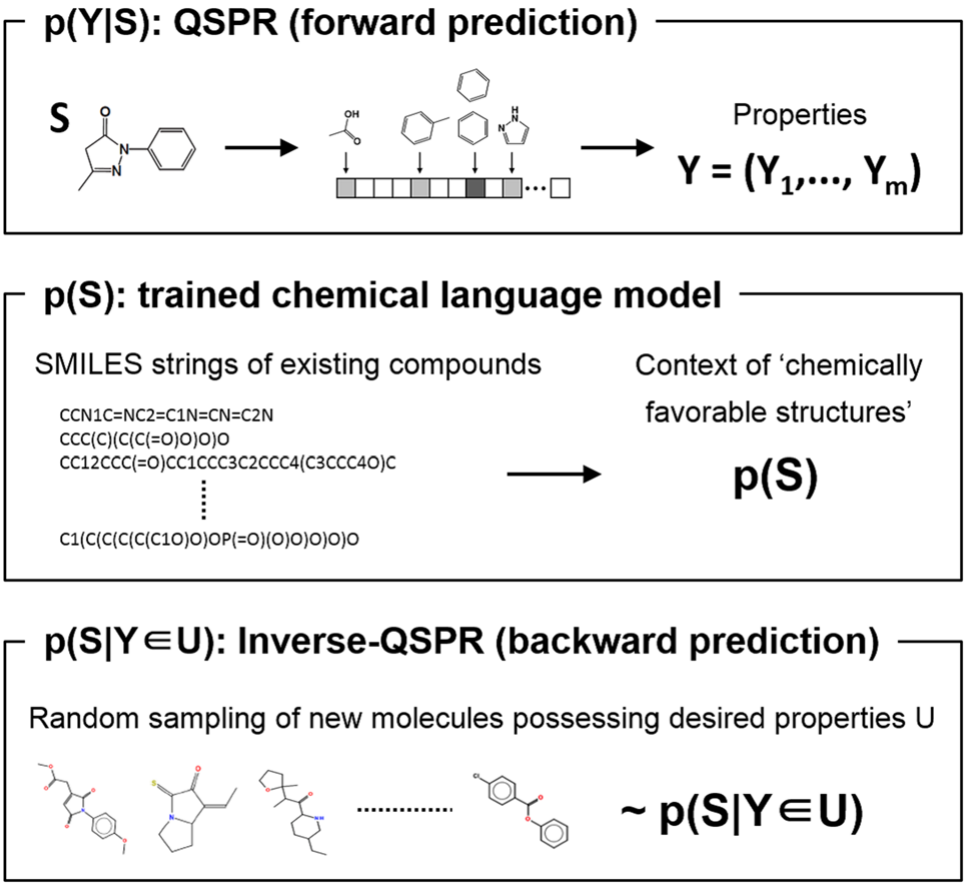
Outline of the Bayesian molecular design method

## Methods

### Outline

The objective of the backward prediction is to create a chemical structure *S* with *p* properties $\mathbf{Y} = (Y_1, \ldots , Y_p)^{\mathrm {T}} \in \mathbb {R}^p$ lying in a desired region *U*. The Bayesian molecular design relies on the statement of Bayes’ law, which is sometimes called the inverse law of conditional probability,

1 $$\begin{aligned} p(S|\mathbf{Y} \in U) \propto p(\mathbf{Y} \in U|S)p(S). \end{aligned}$$

This law states that the posterior distribution $p(S|\mathbf{Y} \in U)$ is proportional to the product of the likelihood $p(\mathbf{Y} \in U| S)$ and the prior *p*(*S*). Exploring high-probability regions of the posterior, we aim to identify promising hypothetical structures *S* that exhibit the desired *U*.

Along with Eq. (1), three internal steps linking the forward and backward analyses are outlined (see also Fig. 1):

- *Forward prediction* A set of QSPR models on the *p* properties is trained with structure-property relationship data. This defines the forward prediction model $p(\mathbf{Y}|S) = \prod _{j=1}^p p(Y_j| S)$ on the right-hand side of Eq. (1).
- *Prior.* The prior distribution *p*(*S*) serves as a *regularizer* that imposes low probability masses on chemically unfavorable structures in the posterior distribution.
- *Backward prediction* Bayes’ law inverts the forward model $p(\mathbf{Y}|S)$ to the backward $p(S|\mathbf{Y} \in U)$ in which a desired property *U* is specified for the conditional. A Monte Carlo calculation is conducted to generate a random sample of molecules $\{S^{r} | r = 1, \ldots , R\}$ of size *R* according to the posterior distribution.

In this study, a chemical structure is described by a SMILES string. As will be detailed, a chemical language model defines the conditional distribution $S' \sim p(S'|S)$ to which the current structure *S* is randomly modified to a new $S'$. By the machine learning of the SMILES language in tens of thousands of existing compounds, structural patterns of real molecules are compressed to the probabilistic language model. In combination with SMC, the trained model, which acquires the implicit meaning of ‘chemically unfavorable structures’, is utilized to modify SMILES strings under a given *U* while reducing the emergence of structures unlikely to occur. Furthermore, the trained language model serves as the prior in Eq. (1).

### Forward prediction

A structure-property data set $\mathcal {D}_j = \{Y_{ij}, S_i: i = 1, \ldots , N\}$ on property *j* is given where $Y_{ij} \in \mathbb {R}^1$ and $S_i$ consist of the *i*th sample. With the *N* observations, a QSPR model is trained by a linear regression $Y_j = \mathbf{w}_j^{\mathrm {T}} \varvec{\psi }_j(S) + \epsilon$ with a *d*-dimensional fingerprint descriptor $\varvec{\psi }_j(S) \in \{0,1\}^d$. To simplify the notation, the property index *j* is temporally omitted. The noise $\epsilon$ is independently and identically distributed according to the normal distribution $\mathrm {N}(\epsilon |0, \sigma ^2)$. The unknown parameters consist of the coefficient vector $\mathbf{w} \in \mathbb {R}^d$ and the noise variance $\sigma ^2 \in \mathbb {R}_+^{1}$. Putting the normal prior $\mathbf{w} \sim \mathrm {N}(\mathbf{w}|\mathbf{0}, \sigma^2 \mathbf{V})$, and the inverse gamma prior $\sigma ^2 \sim \mathrm {IG}(\sigma ^2|a, b)$ on the unknowns, we derive the predictive distribution on the property *Y* with respect to an arbitrary input *S*:

$$\begin{aligned} &p(Y|S, \mathcal {D})= \mathrm {T}_{2 a_*}\Bigg (Y \Big | \mathbf{w}_*^{\mathrm {T}} \varvec{\psi }(S), \frac{b_*}{a_*} (1 + \varvec{\psi }(S)^{\mathrm {T}} \mathbf{V}_* \varvec{\psi }(S))\Bigg ), \\ &\mathbf{V}_*= (\mathbf{V}^{-1} + {\varvec{\Psi }}^{\mathrm {T}}{\varvec{\Psi }})^{-1}, \\& \mathbf{w}_*= \mathbf{V}_* {\varvec{\Psi }}^{\mathrm {T}}{\varvec{y}} \\ &a_*= a + N/2, \ \mathrm {and}\\ &b_*= b + \frac{1}{2} (\mathbf{y} - {\varvec{\Psi }} \mathbf{w}_*)^{\mathrm {T}} (\mathbf{I} + {\varvec{\Psi }} \mathbf{V}{\varvec{\Psi }}^{\mathrm {T}})^{-1} (\mathbf{y} - {\varvec{\Psi }} \mathbf{w}_*), \end{aligned}$$

where ${\varvec{\Psi }}^{\mathrm {T}} = (\varvec{\psi }(S_1), \ldots , \varvec{\psi }(S_N))$ and $\mathbf{y}^{\mathrm {T}} = (Y_1, \ldots , Y_N)$. Here, $\mathbf{I}$ denotes the identity matrix, and $\mathrm {T}_{\nu }(Y | \mu , \lambda )$ denotes the density function of the *t*-distribution with mean $\mu$, scale $\lambda$ and the degree of freedom $\nu$. The predicted value of the property is given by the mean $\mathbf{w}_*^{\mathrm {T}} \varvec{\psi }(S)$ of the predictive distribution.

The prediction models on the *p* properties, $p(Y_j|S, \mathcal {D}_j)$ ($j=1, \ldots , p$), are obtained individually from the respective training sets. We then define the likelihood in Bayes’ law with a desired property region $U=U_1 \times \cdots \times U_p$ as

2 $$\begin{aligned} p(\mathbf{Y} \in U | S) = \prod _{j=1}^p \int _{U_j} p(Y_j | S, \mathcal {D}_j) \mathrm {d} Y_j. \end{aligned}$$

For brevity, we write $p(\mathbf{Y} \in U| S) = p(\mathbf{Y} \in U| S, \mathcal {D})$.

Though a simple instance of QSPR models is described here, we can exploit more advanced techniques of supervised learning such as state-of-the art deep learning or a class of the ensemble learning algorithms. When dealing with a discrete-valued property, the regression should be replaced by a classification model. This study is developed along the use of conventional fingerprints as the descriptor, but it is highly beneficial in practice to use more advanced descriptors, for example, molecular graph kernels coupled with kernel machine learning [23–25].

**Table 1:** Correspondence table between the formal and modified rules of SMILES

Type	Original	Modified
Start of a ring closure	n $$\in \{\mathtt{1}, \mathtt{2}, \ldots \}$$	&
End of a ring closure	n (same to the start)	& $$_i$$ for the *i*th ring terminators to the last of a string
Bond followed by atom *A*	=A (double), #A (triple)	=A or #A form a single character
Terminal character of a molecule	N/A	$
String in a square bracket	[abcde]	[abcde] form a single character

**Fig. 2:**
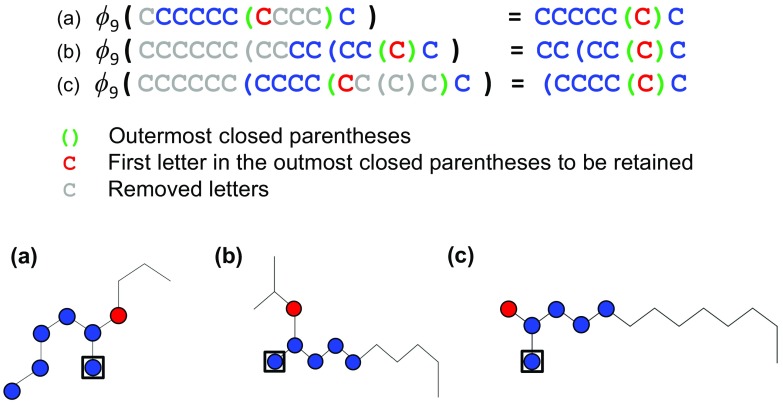
Illustration of the substring selector $\phi _{n-1}(\cdot )$ with three examples. In the contraction operation, a substring inside of the outermost closed parentheses (*green*) is reduced to the character in its first position (*red*). The extraction operation is to remove the rest (*black*) of the last $n-1$ ($=9$) characters from the reduced string. The corresponding graphs are shown on the *right* where the atoms in the *boxes* indicate the last characters in the inputs of $\phi _{n-1}(\cdot )$ (*left*)

### Chemical language model

With the SMILES chemical language, a molecule is translated to a linearly arranged string $$S = s_1 s_2 \ldots s_g$$ of length *g*. A string of the SMILES encoding rules consists entirely of symbols that indicate element types, bond types, and the start and terminal for ring closures and branching components. The start and terminal of a ring closure is designated by a common digit, ‘1’, ‘2’, and so on. A branch is enclosed in parentheses, ‘(’ and ‘)’. Substrings corresponding to multiple rings and branches can be nested or overlapped. In addition to the formal rule of SMILES, all strings are revised as ending up with the termination code ‘$ ’. Inclusion of this symbol is necessary to automatically terminate a recursive string elongation process. For instance, once a string pattern ...CCC=O is present, any further elongation is prohibited and should be terminated at once by appending ‘$ ’. In addition, digits indicating the starts and terminals of rings are represented by ‘&’. The revised representation rule is listed in Table 1. Appendix 1 in Supplementary Materials provides an illustrative example.

With no loss of generality, the prior *p*(*S*) can be expressed as the product of the conditional probabilities:

3 $$\begin{aligned} p(S) = p(s_1) \prod _{i=2}^p p(s_i | s_{1:i-1}). \end{aligned}$$

The occurrence probability of character $s_i$ depends on the preceding $s_{1:i-1} = s_{1} \cdots s_{i-1}$. In general, the non-canonical SMILES encodes a chemical structure into many equivalent forms that correspond to different atom orderings. We treat such structurally equivalent strings as different *S*.

The fundamental idea of the chemical language modeling is as follows: (i) the conditional probability $p(s_{i} | s_{1:i-1})$ is estimated with the observed frequencies of substring patterns in known compounds, and (ii) the trained model is anticipated to successfully learn an implied context of the chemical language. For a given substructure $s_{1:i-1}$, the model is used to modify the rest of the components: until the termination code appears, subsequent characters are recursively added according to the conditional probabilities while putting the acquired chemical reality into the resulting structure.

The SMILES generator should create grammatically valid strings. In particular, we focus on two technical difficulties to be addressed, which are relevant to the rules of grammar on the expression of rings and branching components.

(i) Unclosed ring and branch indicators must be prohibited. For instance, any strings extended rightward from a given $s_{1:6} = \mathtt{CC(C(C}$ should contain two closing characters, ‘)’, somewhere in the rest.
(ii) Neighbors in a chemical string are not always adjacent in the original molecular graph. Consider a structure expressed by CCCCC(CCCCC)C. The substring in the parentheses is a branch of the main chain. The main chain consists of six tandemly arranged carbons that are split into before and after the branch. In this case, the occurrence probability of the final character $s_{13} = \mathtt{C}$ should be affected more by characters in the main chain than those in the branch. In other words, the conditional probability of $s_{i}$ should depend selectively on a preferred subset of the conditional $s_{1:i-1}$ according to the overall context of $s_{1:i-1}$ and $s_{i}$. The same holds when one or more rings appear in the conditional, e.g., c1ccc2ccccc2c1C.

To remedy these issues, the conditional probability is modeled as

4 $$\begin{aligned} p(s_i | s_{1:i-1}) = \prod _{k=1}^{20} p(s_i | \phi _{n-1}(s_{1:i-1}), \mathcal {A}_k)^{I(s_{1:i-1} \in \mathcal {A}_k)}, \end{aligned}$$

where $I(\cdot )$ denotes the indicator function which takes value one if the argument is true and zero otherwise. One of the 20 different models $p(\cdot | \cdot , \mathcal {A}_k)$ ($k = 1, \ldots , 20$) becomes active when the state of the preceding sequence $s_{1:i-1}$ falls into any of the mutually exclusive “conditions” $\mathcal {A}_k$ ($k=1, \ldots , 20$). The 20 ($= 2 \times 10$) conditions are classified according to the presence or absence of unclosed branches and the numbers $\{0, 1, \ldots , 9\}$ of unclosed ring indicators in $s_{1:i-1}$. For instance, if $s_{1:i-1}$ contains two unclosed ring indicators, e.g., CCCC(CC(, the corresponding models should be probabilistically biased toward producing the two terminal characters ‘)’ in subsequent characters. In addition, the substring selector $\phi _{n-1} (s_{1:i-1})$ is introduced for the treatment of the second problem. The definition is as follows:

- *Contraction* Suppose that $s_{1:i-1}$ contains a substring $t = t_1 \cdots t_q$ enclosed by the closed parentheses such that *t* itself is never enclosed by any other closed parentheses. In other words, *t* is a substring inside of the outermost closed parentheses. The substring is then reduced to be $t \rightarrow t' = t_1$ by removing all characters in *t* except for the first character, $t_1$. In other words, $t_1$ is the character that is the right-hand neighbor of the opening ‘(’ of the outermost closed parentheses.
- *Extraction* The selector $\phi _{n-1} (s_{1:i-1})$ outputs the last $n-1$ characters in the reduced string of $s_{1:i-1}$.

The substring selector is illustrated with several examples in Fig. 2. This operation reduces a substring in any nested closed parentheses to a single character that indicates the atom adjacent to the branching point. The occurrence probability of $s_i$ is then conditioned by its $n-1$ preceding characters in the reduced strings that correspond to neighbors in the molecular graph.

Under the maximum likelihood principle, the conditional probability for $\mathcal {A}_k$ in Eq. 4 is estimated by the relative frequency of co-occurring *n*-gram, $s_i$ and $\psi _{n-1}(s_{1:i-1})$, in training instances of known compounds. Let $f_{\mathcal {A}_k}(s_i, \phi _{n-1}(s_{1:i-1}))$ denote the count of the *n*-grams in which the conditional string $s_{1:i-1}$ is in condition $\mathcal {A}_k$. We then conduct the back-off procedure [26] separately with all possible substrings $s_{1:i}$ whose the conditionals $s_{1:i-1}$ belong to $\mathcal {A}_k$:

$$\begin{aligned}&p(s_i | \phi _{n-1}(s_{1:i-1}), \mathcal {A}_k) \\&\quad = {\left\{ \begin{array}{ll} \frac{f_{\mathcal {A}_k}(s_i, \phi _{n-1}(s_{1:i-1}))}{ {\sum _{s_i \in \Sigma }} f_{\mathcal {A}_k}(s_i, \phi _{n-1}(s_{1:i-1})} &{}\quad \mathrm {if} {\displaystyle \sum _{s_i \in \Sigma }} f_{\mathcal {A}_k}(s_i, \phi _{n-1}(s_{1:i-1}))> 0 \\ p(s_i | \phi _{n-2}(s_{1:i-1}), \mathcal {A}_k) &{}\quad \mathrm {otherwise} \end{array}\right. } , \end{aligned}$$

where $\Sigma$ denotes the set of all possible characters. This is a recursive formula across $n = 1, 2, \ldots , n_{\mathrm {max}}$. In the upper formula, the estimate is given by the relative frequency of each instance of an *n*-gram in the $\mathcal {A}_k$-conditioned substrings. If there are no instances, the estimate at the previous $(n-1)$-gram is substituted as in the lower formula.

### Backward prediction

The objective of the backward prediction is to generate chemical strings from the posterior distribution in Eq. (1), conditioned on a desired property region *U*. The forward models and the trained chemical language model define the posterior as in Eqs. (2) and (3). The SMC algorithm that we developed is shown in Algorithm 1.

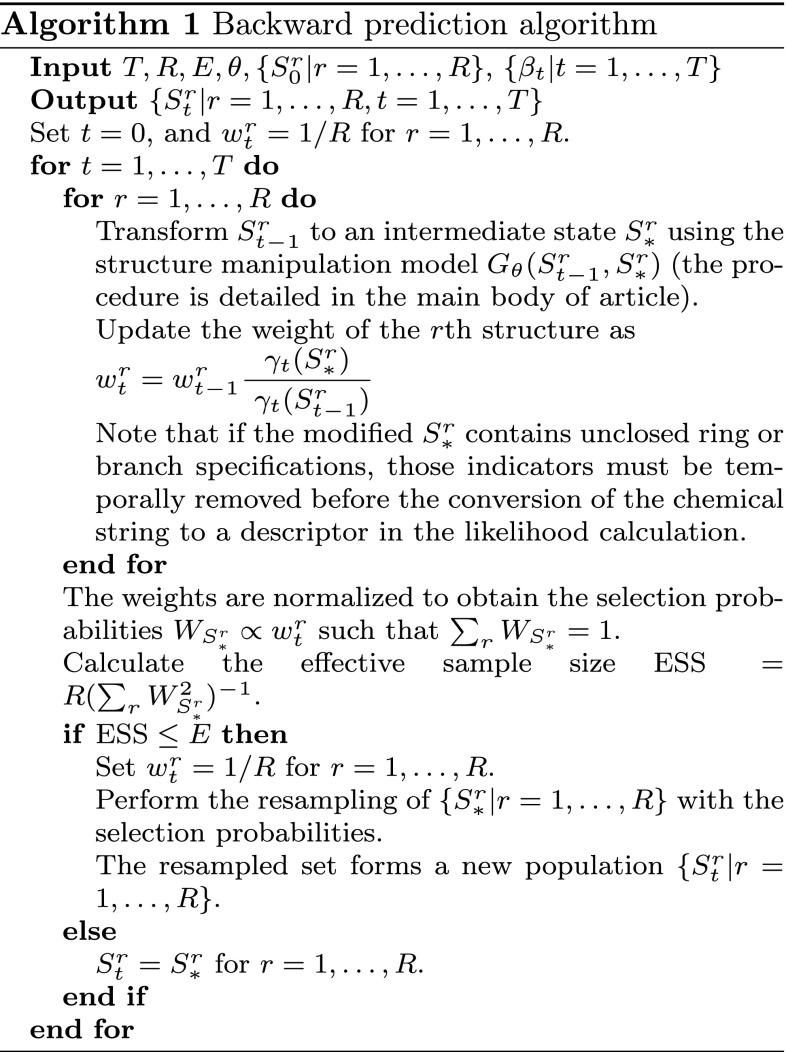



In general, diverse molecules exhibit significantly high probabilities in the posterior. In order to better capture the diversity of promising structures, we create a series of tempered target distributions, $\gamma _t (S)$ ($t=1, \ldots , T$), with a non-decreasing sequence of inverse temperatures $0 \le \beta _1 \le \beta _2 \le \cdots \le \beta _{s-1} \le \beta _s = \cdots = \beta _T = 1$.

$$\begin{aligned} \gamma _t(S) \propto p(\mathbf{Y} \in U | S)^{\beta _t} p(S). \end{aligned}$$

The likelihood function becomes flatter as the inverse temperature decreases, and vice versa. The algorithm begins with a small $\beta _1 \simeq 0$. The series of target distributions monotonically approaches as the iteration number increases, and bridges to the posterior at $\beta _t = 1$, $\forall t \ge s$.

At the initial step $t=0$, *R* structures $\{S_0^{r} | r =1, \ldots , R\}$ are created by some means. For each subsequent *t*, a currently obtained structure $S_{t-1}^{r}$ is mutated randomly to $S_*^{r}$ ($r=1, \ldots , R$) according to a structure manipulation model $G_{\theta }(S_{t-1}^{r}, S_*^{r})$ with a set of parameters, $\theta = (\kappa , \eta )$, as detailed below. A new population $\{S_{t}^{r} | r=1, \ldots , R\}$ is then produced by conducting the resampling of $\{S_*^{r} | r=1, \ldots , R\}$ with the selection probabilities, $W_{S_*^{r}}$ ($r = 1, \ldots , R$), which involve the current tempered distribution $\gamma _t(S)$. The greater the likelihood a mutated structure achieves, the higher the chance it survives and the more the offspring it leaves. In general, this continues until the population has been updated hundreds or thousands of times. The present algorithm is essentially the same as a GA. The crucial difference lies in the mutation operator $G_{\theta }(\cdot , \cdot )$.

The structure manipulation model $G_{\theta }(S, S')$ is designed with the trained SMILES generator as summarized below.

(i) Draw a uniform random number $z \sim \mathrm {U}(0, 1)$. If *S* is grammatically correct and *z* is less than the reordering execution probability $\kappa$ (=0.2), reorder the string $S \rightarrow S^*$ of length *g*, otherwise set the unprocessed string to $S^*$. With the first character chosen randomly using a uniform distribution, Open Babel 2.3.2 [27] is used from the command line with an argument ‘-xf’ for the reordering.
(ii) Discard the rightmost *m* characters of the reordered string to derive $$S^{**} = s_{1:g-m}^{**}$$. The deletion length *m* is sampled from the binomial distribution $$m \sim \mathrm {B}(m|L, \eta )$$ with binomial probability $$\eta$$ (=0.5 by default) and the maximum length *L* (=5 by default).
(iii) Extend the reduced string by sequentially adding a new character to the terminal point $L-m$ times. A newly added character follows the trained language model $s_i \sim p(s_i | s_{1:i-1})$. Once the termination code appears, the elongation is stopped, and then we have $S'$.

The reordering of strings plays a key role in preventing a series of designed molecules from getting stuck in local states. Note that temporally, the SMC algorithm can create structures containing unclosed rings and branching components. Then, the corresponding start codes for the unclosed rings or branches are temporally removed to avoid the syntax error when obtaining a descriptor for the likelihood calculation. In addition, the atom order is rearranged only when a current string is grammatically valid.

### Software

The *iqspr* package can be installed thorough the CRAN repository. Installation of Open Babel 2.3.2 is required for getting started. The package consists of a set of functions to perform the QSPR model building (*QSPRpred reference class*) with molecular fingerprints in the *rcdk* package [28], the inverse-QSPR prediction (*SmcChem reference class*), and the training and simulation of the chemical language generator (*ENgram reference class*) with user-specified input SMILES strings. Currently, the chemical language modeling and the inverse analysis cannot deal with isomers, or ionic compounds. A sample code is given in Appendix 2 in Supplementary Materials.

**Table 2:** MAEs of the QSPR models with the eight different fingerprint descriptors for the internal energy and the HOMO-LUMO gap

Fingerprint	Energy (kcal/mol)	HOMO-LUMO gap (eV)	Runtime (s)
1	32.6	0.53	0.50
2	30.4	0.54	0.41
3	29.3	1.37	2.57
4	28.3	1.66	0.36
5	22.1	0.55	5.32
6	46.8	0.84	0.39
1,2,4	23.5	0.54	1.61
1,2,4,5	18.9	0.50	7.71

The six fingerprints in the *rcdk* package (bottom) and their combinations were tested. The last column denotes the average runtime for the QSPR score (likelihood) calculation per 100 molecules. The runtimes were measured on an Intel Xeon 2.0 GHz processor with 128 GB memory using the *iqspr* package

1. ‘standard’: paths of a default length (1024 bits)

2. ‘extended’: the ‘standard’ fingerprint is modified such that ring and atomic properties are taken into account (1024 bits)

3. ‘maccs’: MDL MACCS keys (166 bits)

4. ‘circular’: ECFP6 fingerprint (1024 bits)

5. ‘pubchem’: PubChem fingerprint (881 bits)

6. ‘graph’: ‘standard’ is modified by taking into account connectivity (1024 bit)

## Results and discussion

### Data set

The molecular design process is demonstrated through the creation of small organic molecules with the design objective intended to the HOMO-LUMO gap ($\mathrm {HL}$) [29, 30] and the internal energy ($\mathrm {E}$). With the quantum chemistry calculation based on DFT, the two properties were obtained for 16,674 chemical instances which were selected randomly from PubChem [31] (available at Supplementary Data 1). The data set does not contain molecules including one or more inorganic elements which are surrounded by square brackets in the SMILES notation, or atomic symbols with chiral specification @. Strings including either ‘+’ or ‘-’, representing ionic elements, were also excluded. Because of its performance and suitability for our parallel computer facilities, the Gaussian09 suite of program codes [32] was used to carry out all the present DFT simulations. The corresponding molecular structures were obtained via the PubChemQC project [33], which were fully optimized at the B3LYP/6-31+G(d) level of theory though using GAMESS [34, 35]. We found that further optimization at the same level with Gaussian09 leaves the GAMESS geometries unchanged for some preliminary cases. Therefore, the two target properties were evaluated for all the cases from the single-point B3LYP/6-31+G(d) calculations.

In the forward analysis, the entire set was divided into 10,000 and 6674 instances for training and testing, respectively. The chemical language model was trained with 50,000 organic compounds without chiral specification @ or ionic elements ‘+’ and ‘-’, which were selected randomly from the PubChem database. The performance of the backward prediction was tested on three different property regions of $\mathbf{Y} = (Y_{\mathrm {HL}}, Y_{\mathrm {E}})^{\mathrm {T}}$: (i) $U_1 = [100, 200] \times [4, 5.5]$, (ii) $U_2 = [250, 400] \times [5, 6]$, and (iii) $U_3 = [100, 250] \times [2.5, 3.5]$. Designed hypothetical molecules were validated with the DFT calculation as to whether or not their physical properties fall within each desired range.

### Forward prediction

As shown in Table 2, eight different descriptors ${\varvec{\psi }}(S)$ were derived by using six types of molecular fingerprints in combination; which these fingerprints are implemented in the R package *rcdk* [28]. The mean of the predictive distribution was employed as the predicted value of each property. The parameters of the normal and gamma priors in regression were set as ${\mathbf V} = {\mathbf I}$ and $(a, b) = (0, 0)$. The performance of the trained models was assessed with the mean absolute error (MAE). As shown, the augmented descriptor that combined the ‘standard’, ‘extended’, ‘circular’ and ‘pubchem’ fingerprints delivered the highest predictive accuracy. However, the average runtime for the likelihood calculation per 100 molecules ($\sim$7.71 s) was significantly greater than the others because the translation into the PubChem fingerprint involves an intractable graph pattern matching. This led to a significant increase in the runtime of the backward prediction. We therefore employed the second-best descriptor containing ‘standard’, ‘extended’ and ‘circular’, which delivered relatively small MAEs, 0.54 eV and 23.5 kcal/mol, for the HOMO-LUMO gap and internal energy, respectively. With this, the runtime was reduced by nearly $80\%$ (to $\sim$1.61 s per 100 molecules), compared with the best performing model.

**Fig. 3:**
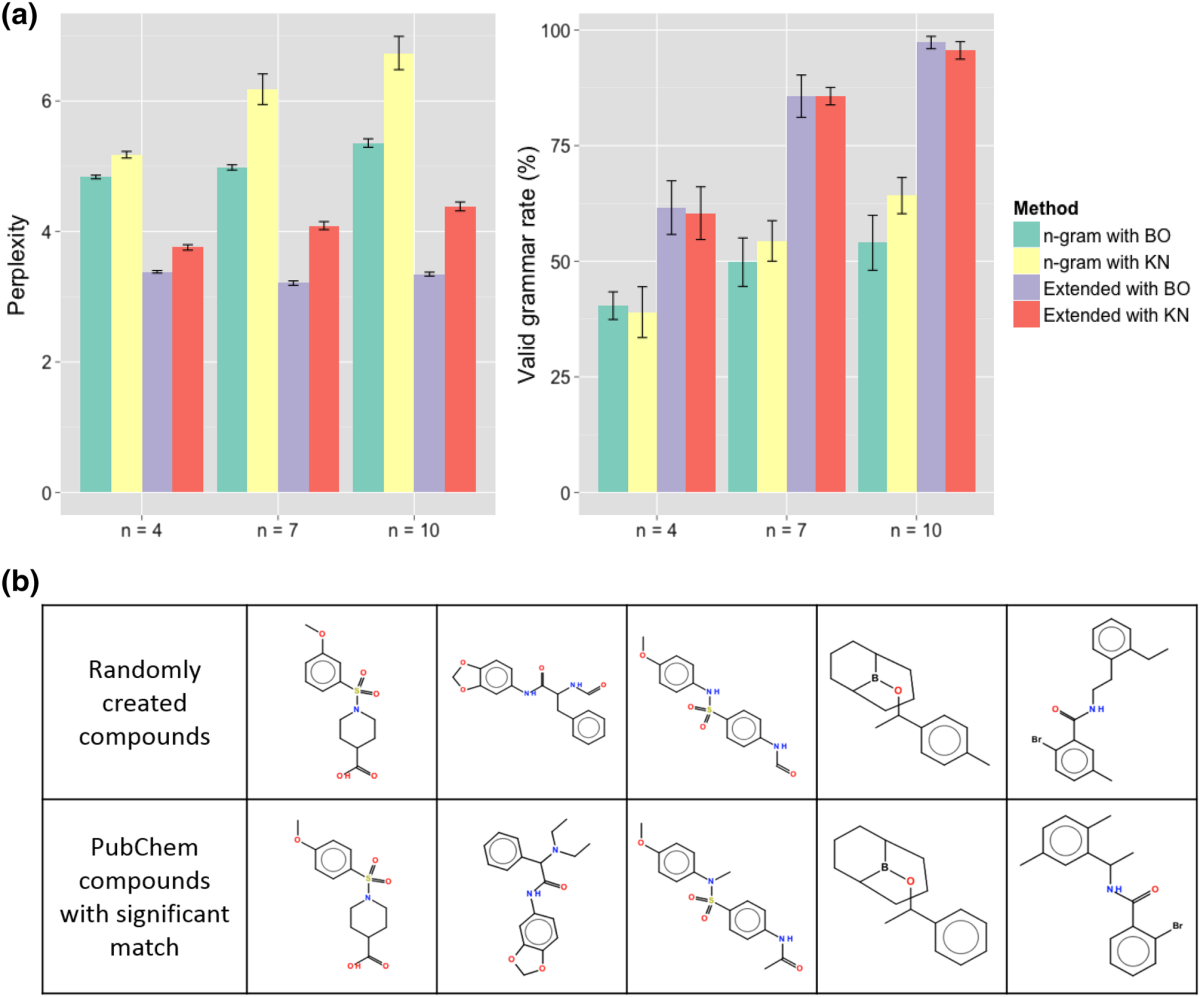
**a** Perplexity scores (*left*) and valid grammar rate (1 − the syntax error rate) (*right*) with respect to 1000 SMILES strings generated from trained chemical language models. The conventional *n*-gram and the extended language models were trained with the BO and KN algorithms. The *error bars* represent the standard deviations across the 10 experiments corresponding to different training sets. **b** Examples of molecules generated from the trained chemical language model with $n=10$ (*top*). The *bottom row* displays the most similar PubChem compounds that had the Tanimoto coefficient $\ge$0.9 on the PubChem fingerprint

**Table 3:** Parameters and experimental conditions for the Bayesian molecular design analysis

Process	Description	Parameter
Forward prediction	Number of training data	$N = 10{,}000$
	Fingerprint descriptor	1, 2, 4
	The normal prior	$\mathbf{V} = \mathbf{I}$
	The Gamma prior	$(a, b) = (0 , 0)$
Chemical language model	Number of training data	50,000
	Markov-order	$n = 10$
	Estimation algorithm	Back-off method
Backward prediction	Size of population	$R = 100$
	Number of iterations	$T = 500$
	Reordering probability	$\kappa = 0.2$
	Binomial probability	$\eta = 0.5$
	Trial number	$L = 0.5$
	Cooling schedule	$\beta _t = 5^{0.95^{t-1}}$ for $t \le 250$, $\beta _t = 1$ for $t \ge 251$
	Threshold on ESS	$E = 50$
	Initial structures	Phenol c1ccccc1O

### Chemical language model

To determine the order *n* of the chemical language model and to verify its learning ability in the chemical language context, ten training sets of 1000 compounds were randomly produced from the PubChem compounds. Each set was halved for training $\mathcal {D}_{\mathrm {train}}$ and testing $\mathcal {D}_{\mathrm {test}}$. The selected model was learned all over again with 50,000 different training compounds for the inverse-QSPR prediction.

The models with varying orders, $n \in \{4, 7, 10\}$, were trained with two different procedures, the back-off (BO) and the Kneser–Nay smoothing (KN) methods [26]. As a control group in the comparison, we added a conventional *n*-gram that learned the $(n-1)$-order Markov relationship among the chemical strings simply without using the stratification $\mathcal {A}_k$ ($k=1, \ldots , 20$) and the substring selector $\phi _{n-1}(\cdot )$. Model performances were evaluated with two criteria: the perplexity measure [36] and the grammatical validity of produced chemical strings.

Perplexity is a commonly used measure in the natural language processing that evaluates the generalization capability of a language model $\mathcal {M}$ with the trained probability function $p_\mathcal {M}(S)$ in Eq. 3,

$$\begin{aligned} \mathrm {perplexity}(\mathcal {M}) = \exp \Big ( - \frac{1}{| \mathcal {D}_{\mathrm {test}}|} \sum _{i \in \mathcal {D}_{\mathrm {test}}} \log p_\mathcal {M} (S_i) \Big ). \end{aligned}$$

For each model, the goodness-of-fit, i.e., the likelihood, to the 1000 test instances was measured. As shown in Fig. 3a, the models resulting from BO outperformed the others in terms of perplexity. In the comparison among the BO-derived models with the different orders, there were no significant differences in the generalization capability. Furthermore, this experiment showed the significance of the stratification $\mathcal {A}_k$ ($k = 1, \ldots , 20$) and the substring selector $\phi _{n-1}(\cdot )$, as significant improvements of perplexity were observed in the extended models relative to the conventional models.

In light of grammatical validity, the syntax error rates were evaluated for 1000 hypothetical molecules generated from each of the ten trained models. The grammar check was done with the SMILES parser function ‘parse.smiles’ in the *rcdk* package with the option ‘kekulise = TRUE’. As shown in Fig. 3a, the error rate was monotonically reduced with an increase in the Markov order in the extended models. The minimum error rate ($\le$2.7 %) was attained at $n = 10$. The performances of the BO and KN algorithms were much the same. In conclusion, we selected the BO-derived model with $n = 10$ based on perplexity and grammatical validity.

To further validate the learning ability of the BO-derived model with $n = 10$, randomly created 50 molecules were associated with PubChem compounds in which the training compounds were removed. Approximately $72\%$ of the 50 virtual molecules exhibited extensive similarities to one or more existing compounds meeting the acceptance criterion of the Tanimoto coefficient $\ge$0.9 on the PubChem fingerprint. Figure 3b shows five instances of the created molecules; these instances indicate the great ability of the chemical language model. Conventional structure generators could never reproduce such structurally complex molecules.

**Fig. 4:**
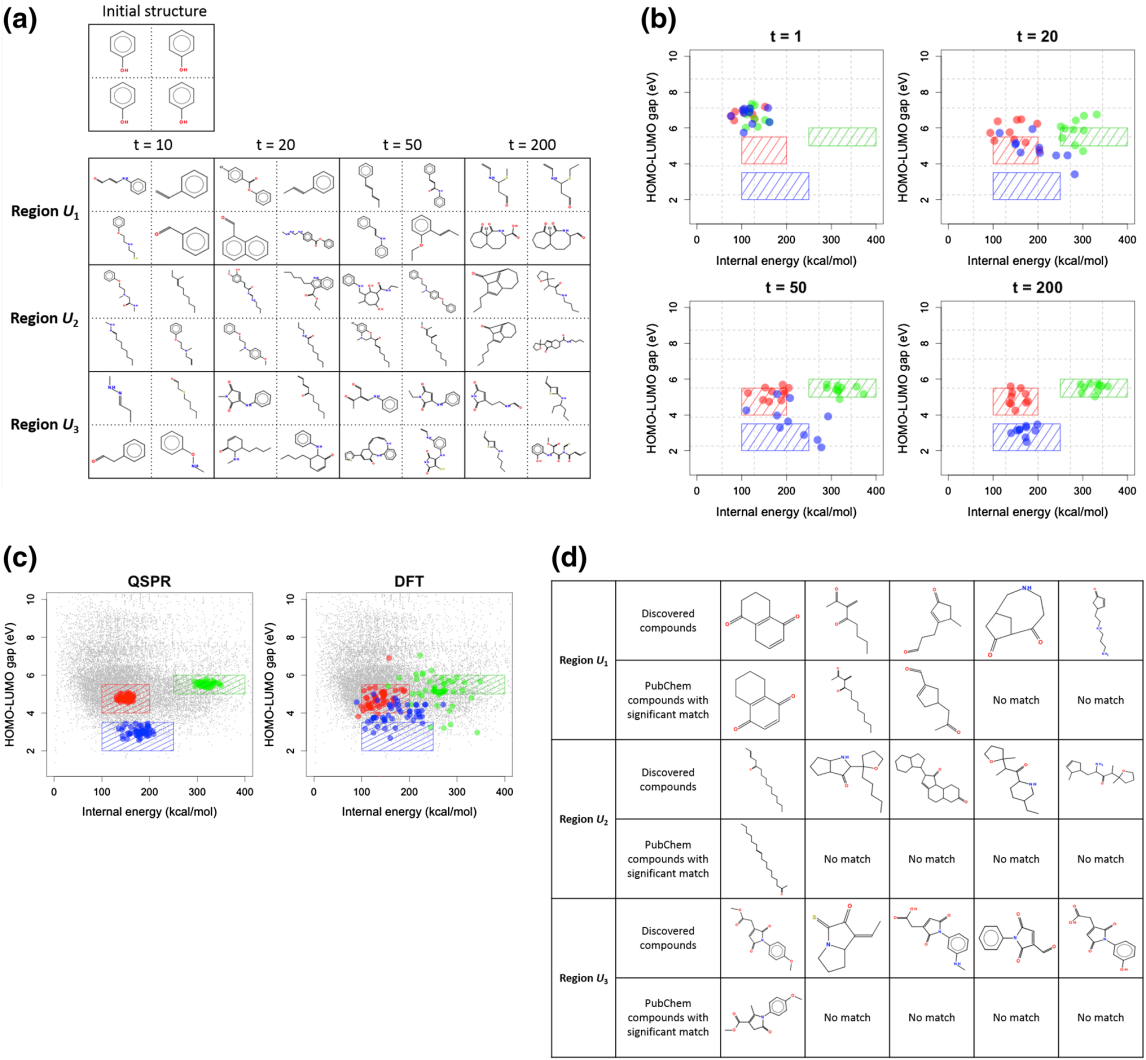
**a** Snapshots of structure alteration during the early phase of the inverse-QSPR calculation ($t \in \{10, 20, 50, 200\}$) with the desired property region set to $U_1$, $U_2$ or $U_3$. The initial molecule (phenol) is shown at the *top*. The created molecules shown here were those ranked in the top four by the likelihood score at each *t*. Supplementary Movie 1–3 visualize the whole processes of structure modification over $t \in [1, 200]$. **b** Property refinements resulting from the backward prediction at $t \in \{1, 20, 50, 200\}$. Results on the three different property regions, $U_1$, $U_2$ and $U_3$, are displayed together, and color-coded by *red*, *green* and *blue*, respectively. The *shaded rectangles* indicate the target regions. The *dots* indicate the HOMO-LUMO gaps and internal energies of the designed molecules that were calculated by the predicted values of the QSPR models. For each $U_i$ and *t*, the 10 non-redundant molecules exhibiting the greater likelihoods are shown. **c** Properties of 50 molecules which were selected from the overall backward prediction process for $U_1$ (*red*), $U_2$ (*green*), and $U_3$ (*blue*). The HOMO-LUMO gap and internal energy were calculated by the trained QSPR models (*left*) and the DFT calculation (*right*). The *gray dots* indicate the training data points. In each $U_i$, the 50 non-redundant molecules that achieved the highest likelihoods are shown. **d** Newly created molecules in the predefined property regions. The *bottom row* of each pair shows instances of significantly similar PubChem compounds that had the Tanimoto index $\ge 0.9$

### Backward prediction

Table 3 summarizes the parameters of the backward prediction. Phenol ’c1cccccc1O’ was assigned to the 100 initial structures ($R = 100$) which were refined across $t = 1, \ldots , T$ with $T=500$ as a desired property region was sought. The movies in Supplementary Movie 1–3 show the processes of transforming structures aimed at the given property regions, $U_1$, $U_2$ and $U_3$, respectively. Figure 4a shows snapshots of these processes. The created molecules underwent substantial changes in size, geometry and composition. A visual inspection of the movies verifies that backward calculation prevents structures from getting stuck in locally high-probability regions.

Figure 4b illustrates the early stages ($t \in \{1, 20, 50, 200\}$) of the property refinements, during which they are moving in toward their respective target regions. For each *t*, a non-redundant set of created molecules is shown: molecules ranked in the top 10 by the likelihood score were selected from a ranking list in which a molecule was removed from the list if its Tanimoto coefficient on the PubChem fingerprint exceeded 0.9 with respect to any of the higher ranking molecules. The reported HOMO-LUMO gap and internal energy correspond to the means of the predictive distributions for the trained forward models. At $t=1$, the properties were very far from the desired regions. As the calculation proceeds, the resulting properties approached the targets quite rapidly. At $t=200$, almost all of the created molecules had properties falling within their respective target region, $U_1$, $U_2$, or $U_3$. This observation indicates that the proposed method is capable of drastic and rapid refinements of the properties of seed molecules.

Figure 4c shows the properties of molecules created at $t=251$ and 500 with their verifications by the DFT calculation. In the same way described above, 50 non-redundant molecules were selected from the likelihood-based prioritized list of 25,000 candidates: similar to the results shown in Fig. 4b, 50 non-redundant molecules were selected, in this case selected from a prioritized list of the 25,000 candidates corresponding to the 100 particles produced between $t=251$ and 500. The physical properties were evaluated by the QSPR models and the DFT calculation. For the DFT calculation, the created SMILES strings were first converted into the 3D structures by using OpenBabel with the ‘-gen3d’ option. Such initial conformations were fully optimized using Gaussian09 with B3LYP/6-31+G(d). Finally, the electronic properties at the equilibrium geometries were computed at the same level of theory. As shown, all the QSPR-derived properties of the created molecules fell within the respective desired regions. However, in the verification by the DFT calculation, the arrival rates for $U_2$ and $U_3$ were significantly reduced to 25/50 and 7/50, while the high rate (45/50) was maintained on $U_1$. The cause of the performance depression in the former cases is apparent. As shown in Fig. 4c, the number of known compounds used for the training was fairly small in neighborhoods of $U_2$ and $U_3$. By necessity, the trained forward models had much lower accuracies in prediction in neighborhoods of $U_2$ and $U_3$ relative to $U_1$. The ability of the backward prediction therefore declined as the desired properties were placed within regions where data are sparsely populated. The proposed method has a great ability to discover molecules when a desired property lies within a region where enough data are given, but the creation of truly novel molecules that reside in a far tail of the distribution of known molecules is an issue yet to be addressed. This will be discussed more in the “Concluding remarks” section.

The novelty of derived molecules was investigated by seeking structurally similar compounds in PubChem. For a created *S* that appeared in $U_i$ in terms of DFT, we calculated the Tanimoto coefficient $T(S, S^*)$ on the PubChem fingerprint with respect to all PubChem compounds $S^*$ after removing the training instances. Under the acceptance criterion $T(S, S^*) \ge 0.9$, significantly similar known compounds were identified for *S*. Figure 4d illustrates an instance of promising hypothetical molecules and the results of the similarity search. Thus, it has been confirmed that the proposed method can reproduce the highly complex and diverse molecules in the database. As expected, molecules that emerged in $U_2$ and $U_3$ were less well matched to existing compounds. More importantly, it has been proved that various types of molecules can exist in the same property region and that many of these have yet to be identified. In practice in science and industry, such molecules could be truly important candidates for further testing and synthesis.

The backward prediction algorithm was run on an Intel Xeon 2.0 GHz processor with 128 GB memory using the *iqspr* package. The average execution time was about five seconds per step in SMC. The essential part of the current implementation was all developed in the R language and does not support parallel processing. The development of more advanced software is a future subject.

## Concluding remarks

This study presented a principled approach to computational molecular design thorough a unified Bayesian perspective to the forward and backward predictions in the structure-property relationship analysis. The method was demonstrated with multi-objective molecular design for the prescribed regions of the HOMO-LUMO gap and internal energy. The presented analyses can be performed with the R package *iqspr* that we developed. The structure-property data set generated from the high-throughput DFT calculation has been made available online. Despite potentially great impacts on science and industry, the use of computer-aided molecular design methods has not been widely adopted. The lack of easy-to-access software and benchmark data has restrained the proliferation of the use of inverse-QSPR and the growth of methodologies and tools has been hampered due to the difficulty of performance competition.

The main contribution of this study lies in the newly developed structure refinement algorithm based on the chemical language model. As mentioned earlier, most existing methods utilize chemical fragments of real compounds for the reduction of creating chemically unfavorable molecular graphs. The drawback of the fragment-based methods is the limited diversity of the created structures. To enhance diversity and novelty, a vast number of fragments should be used, but this makes the operation of structure transformation in the fragment exchange process and similarity search on the large fragment library much more computationally expensive. The present study showed the great promise of a fragment-free strategy based on a chemical language model. The trained model acquired the implicit meaning of ‘chemically favorable structures’ and succeeded in the creation of seemingly realistic molecules. Surprisingly, more than $70\%$ of the generated molecules had significantly similar known compounds, and in addition, some of these were structurally very complex to the point that no conventional structure creators would ever be able to reproduce them. The proposed method demonstrated a new way to make computationally efficient structure refinements based on the string representation of molecules. It is important to see that the acquired context of the chemical language is not well defined, but rather is ambiguous. Possibly, the trained language model did not recognize higher-level chemical knowledge such as chemical stability, synthesizability, and drug-likeliness. The creation of much more realistic and valid structures is an important consideration in future work. It should be remarked that more recently, a research group has proposed a molecular generator that relies on a neural network trained on SMILES instances of real molecules [37]. This generator was designed to achieve the same purpose as our study.

As demonstrated, the backward method is enormously powerful in the exploration when enough data are observed in a neighborhood of a specified property region. However, the prediction ability declines as the desired properties are placed around regions where data are sparsely populated. The ultimate goal of computational molecular design is the creation of truly novel molecules that reside in an exceedingly far tail of the distribution of known molecules. The apparent cause of the limited ability is that the trained forward models become less accurate in property prediction in far tails of the training set. This is an issue common to all existing methods but less attention has been paid to this important problem. Ultimately, we wish to arrive in yet-unexplored property regions where no one has gone before. In Supplementary Fig. 1, we have provided snapshots of the property refinement process that explored a yet-unexplored property region, to emphasize the significance of overcoming this limitation. Within early steps, the resulting properties approached the desired region quite rapidly, but the search trajectories became more disperse as they got closer to the target.

A promising solution to this problem might be the integration of computer experiments and the backward prediction algorithm with experimental design techniques. Once created molecules get fairly close to an unexplored property region, a new set of structure-property data could be produced in a neighborhood of the region by conducting, for instance, a first-principle calculation with respect to a preferred subset of the currently created structures. Then, one could refine the forward models using the newly added data. Possibly, the query points of the computer experiment should rationally be selected under a sequential design strategy by maximizing the expected improvement of prediction under a given constraint of computational costs. The refined backward prediction might acquire a greater ability to move a step closer to the target region. The integration of the backward prediction algorithm and rationally designed adaptive data production is the next challenge in future work.

## Electronic supplementary material

Below is the link to the electronic supplementary material.

Supplementary material 1 (PDF 179 kb)

Supplementary material 2 (CSV 793 kb)

Supplementary material 3 (MP4 9088 kb)

Supplementary material 4 (MP4 10824 kb)

Supplementary material 5 (MP4 11932 kb)
