# Supplementary material for: Bayesian molecular design with a chemical language model
Source: J Comput Aided Mol Des. 2017 Mar 9;31(4):379–91. doi: 10.1007/s10822-016-0008-z (PMC5393296; doi:10.1007/s10822-016-0008-z)
Supplement: Supplementary file 1 — Supplementary material 1 (PDF 179 kb) [file 10822_2016_8_MOESM1_ESM.pdf]

Supplementary Materials

Bayesian molecular design with a chemical language model

Hisaki Ikebata[1] Kenta Hongo[2,3,4] Tetsu Isomura[5] Ryo Maezono[2] Ryo Yoshida[1,3,6]

Appendix 1: Revision of SMILES representation rule

Table 1 in the main body of the article summarizes the correspondence between the formal SIMILES and the revised encoding rule. For example, a SMILES string of a molecule, $T =$`c12CNC(Cc1nc[nH]2)C(=O)O`, is revised to $S =$`c&&CNC(Cc&`$_2$`nc[nH]&`$_1$`)C(=O)O$` as $s_1 =$ `c`, $s_2 =$ `&`, $s_3 =$ `&`, $s_4 =$ `C`, $s_5 =$ `N`, $s_6 =$ `C`, $s_7 =$ `(`, $s_8 =$ `C`, $s_9 =$ `c`, $s_{10} =$ `&`$_2$, $s_{11} =$ `n`, $s_{12} =$ `c`, $s_{13} =$ `[nH]`, $s_{14} =$ `&`$_1$, $s_{15} =$ `)`, $s_{16} =$ `C`, $s_{17} =$ `(`, $s_{18} =$ `=O`, $s_{19} =$ `)`, $s_{20} =$ `O`, $s_{21} =$ `$`. The molecule contains two rings indicated by the two digits `1`' and '2' in T. These characters are revised to the start and terminal characters '`&`' and '`&`$_i$' ($i \in \{1, 2\}$). The bracket-surrounded characters `[nH]` in T form the single character $s_{13} =$ `[nH]` in S. Also, the bond followed by '`O`' is concatenated with that the right-hand adjacent atom as $s_{18} =$ `=O`. Finally, the character '`$`' appears at the end of S to indicate that the structure is fully defined.

Appendix 2: Sample code of *iqspr* package

```
#install.packages("iqspr ")#Install package
library(iqspr) #Call package

#Forward prediction
data(qspr.data) #sample data
idx <- sample(nrow(qspr.data), 5000)#Selection of training data

#SMILES of training data for regression
smis <- paste(qspr.data[idx ,1])

#Property (HOMO-LUMO gap, internal energy)
y <- qspr.data[idx , c(2,5)]

#Bayesian linear regression (descriptor: "graph")
qsprpred <- QSPRpred$new(
                    smis=smis, y=as.matrix(y), v_fpnames="graph")

#Prior distribution with the chemical language model
data(trainedSMI) #Training data
engram <- ENgram$new(trainedSMI, order=6) #6-gram model

#Specification of target regions for the properties
# (min and max of internal energy and HOMO-LUMO gap)
qsprpred$ymin <- c(200, 1.5)
qsprpred$ymax <- c(350, 2.5)
```

Ryo Yoshida
10-3 Midori-cho, Tachikawa, Tokyo 190-8562, Japan
[1] The Graduate University for Advanced Studies (SOKENDAI), [2] Japan Advanced Institute of Science and Technology (JAIST), [3] National Institute for Materials Science (NIMS), [4] PRESTO, Japan Science and Technology Agency (JST), [5] The KAITEKI Institute, Inc., [6] The Institute of Statistical Mathematics (ISM), Research Organization of Information and Systems

```
#Backward prediction (initial structure = phenol)
smchem <- SmcChem$new(smis=rep("c1ccccc1O", 25),
                   v_qsprpred=qsprpred,
                   v_engram=engram, temp=3, decay=0.95)
                   smchem$smcexec(niter=100, preorder=0, nview=4)
gensmi <- get_hiscores(smchem, nsmi=4)
viewstr(gensmi[1:4, 1])
```

Supplementary Figure 1

Snapshot for the process of refining the HOMO-LUMO gap and internal energy (blue dots) toward a desired region (rectangle) where training data (gray dots) are sparsely populated.

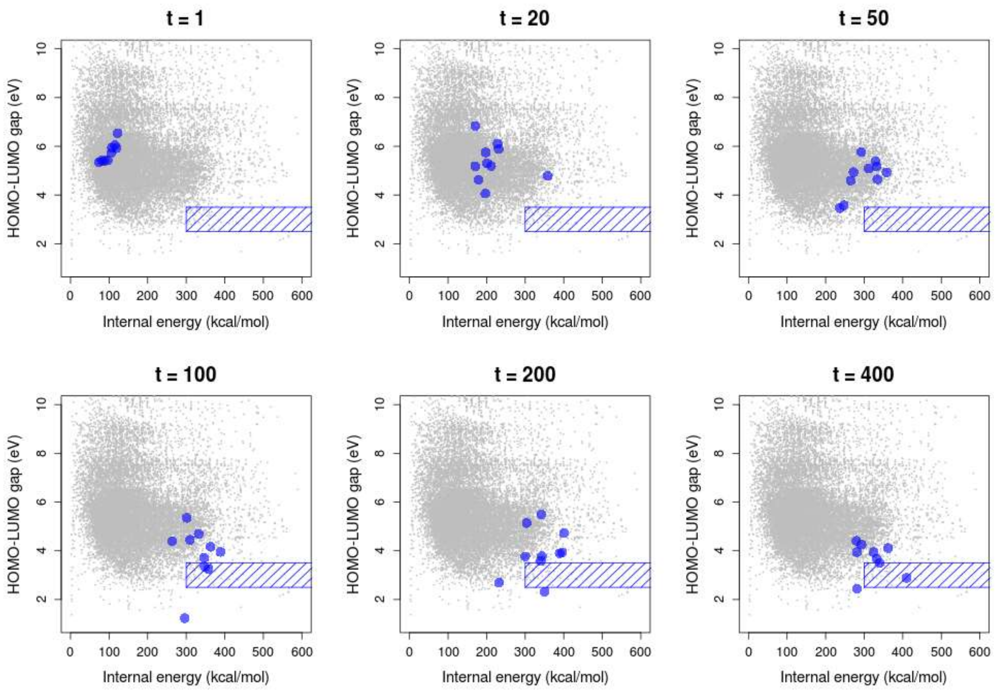

Supplementary Data 1

Structure-property data set, available at the supporting information website. The HOMO-LUMO gaps and internal energies of 16,674 instances of organic compounds in PubChem were calculated by DFT with GAUSSIAN09.

Supplementary Movie 1

Movie of the process of transforming structures with the desired property region U_1, available at the supporting information website.

Supplementary Movie 2

Movie of the process of transforming structures with the desired property region U_2, available at the supporting information website.

Supplementary Movie 3

Movie of the process of transforming structures with the desired property region U_3, available at the supporting information website.
